# BPI-MVQA: a bi-branch model for medical visual question answering

**DOI:** 10.1186/s12880-022-00800-x

**Published:** 2022-04-29

**Authors:** Shengyan Liu, Xuejie Zhang, Xiaobing Zhou, Jian Yang

**Affiliations:** 1 Kunming Shipborne Equipment Research and Test Center, Kunming, 650106 People’s Republic of China; 2 School of Information Science and Engineering, Yunnan University, No. 2, North Cuihu Road, Kunming, 650091 People’s Republic of China

**Keywords:** VQA-Med, Transformer, Parallel structure model, Image retrieval model, Multi-head attention mechanism

## Abstract

**Background:**

Visual question answering in medical domain (VQA-Med) exhibits great potential for enhancing confidence in diagnosing diseases and helping patients better understand their medical conditions. One of the challenges in VQA-Med is how to better understand and combine the semantic features of medical images (e.g., X-rays, Magnetic Resonance Imaging(MRI)) and answer the corresponding questions accurately in unlabeled medical datasets.

**Method:**

We propose a novel Bi-branched model based on Parallel networks and Image retrieval for Medical Visual Question Answering (BPI-MVQA). The first branch of BPI-MVQA is a transformer structure based on a parallel network to achieve complementary advantages in image sequence feature and spatial feature extraction, and multi-modal features are implicitly fused by using the multi-head self-attention mechanism. The second branch is retrieving the similarity of image features generated by the VGG16 network to obtain similar text descriptions as labels.

**Result:**

The BPI-MVQA model achieves state-of-the-art results on three VQA-Med datasets, and the main metric scores exceed the best results so far by 0.2$\%$, 1.4$\%$, and 1.1$\%$.

**Conclusion:**

The evaluation results support the effectiveness of the BPI-MVQA model in VQA-Med. The design of the bi-branch structure helps the model answer different types of visual questions. The parallel network allows for multi-angle image feature extraction, a unique feature extraction method that helps the model better understand the semantic information of the image and achieve greater accuracy in the multi-classification of VQA-Med. In addition, image retrieval helps the model answer irregular, open-ended type questions from the perspective of understanding the information provided by images. The comparison of our method with state-of-the-art methods on three datasets also shows that our method can bring substantial improvement to the VQA-Med system.

## Background

The visual question answering in medical domain (VQA-Med) system has great potential in medical applications, but it is not yet well developed. The original medical question and answer (QA) system was developed prior to VQA-Med and was mainly used for information retrieval, databases, and other technologies. The representative works are the MedQA [1], MiPACQ [2], and AskHERMES [3] systems. Current medical QA systems are generally based on knowledge mapping technology, which stores medical information in the form of an entity-relationship in a non-relational database, and they provide medical advice by searching and reasoning, Aarthi [4] enumerates the traditional subtasks of QA, including almost all MedQA questions. For example, Izcovich [5] developed a GRADE-based medical question answering system. However, the ability to analyze medical test cases is not sufficient for clinical adoption; analyzing medical images is also a necessary skill of the auxiliary medical system. A VQA system [6] can meet this requirement. Such a system utilizes computer vision (CV) and natural language processing (NLP) to systematically learn the features of given images and questions and then generates answers to the questions. At first, VQA technology was widely used for fine-grained recognition, object recognition, and behavior recognition in random scenes including people or objects. These tasks require the VQA system to not only classify images and detect targets, but also to extract semantic features and have a certain degree of common sense. When the ImageCLEF2018 competition [7] proposed a VQA-Med task in 2018, VQA was applied to the medical field for the first time. Similar to VQA, the questions of VQA-Med include organ type recognition (e.g., what organ is this?), abnormal type identification (e.g., is the lung abnormal?), and classification of medical images (e.g., what is the imaging mode of given medical images?). Due to the lack of annotation information in medical datasets—such as the labeling of organ lesions and the center point, length, and width of the boundary box of the location of lesions—we cannot use a series of effective target detection methods in the field of general VQA to help extract medical image features, which makes it difficult to apply VQA in specific fields. Visual Question Generation (VQG) from images is also a rising research topic in both fields of natural language processing and computer vision [8]. Although there are some recent efforts towards generating questions from images in the open domain, it also represents another meaningful solution to the VQA-Med task.

With the emergence and popularization of new digital medical imaging equipment [9], clinicians can use both knowledge and medical equipment to diagnose diseases. In some cases of non-obvious trauma, medical imaging is much more informative than patient-reported symptoms. However, interpreting medical imaging is challenging for inexperienced interns and medical students. A well-established VQA-Med system can help them practice and judge whether their conclusions are correct or not. Traditional computer-aided diagnosis technology is usually aimed at one disease; for example, for judging the probability of lung cancer based on the presence of pulmonary nodules in Computed Tomography(CT) images of the chest [10], for detecting tuberculosis and classifying its severity [11], or for detecting breast cancer based on chest radiographs [12]. A major limitation of auxiliary diagnosis technology based on analyzing a single type of medical imaging is its inability to provide a complicated, specific description of a patient’s condition similar to a clinician’s diagnosis. The VQA-Med system can realize this function. However, the current VQA-Med datasets generally have substandard problems, which is a part that needs to be improved, because even if there is a large amount of training data support, wrong data will lead to an increase in classification inaccuracy. VQA-Med has great research significance. First of all, it is in its infancy, and there are still many technologies to be explored. Secondly, because of the lack of standardized data sets, we need to make the model have good data adaptability. Based on the model research foundation of VQA-Med, a series of methods in this paper are proposed for VQA-Med, which makes the VQA-Med system convenient for patients’ consultation and doctors’ research. In addition, VQA-Med also faces many challenges, such as special processing of medical-specific vocabulary in medical texts and medical images, the problem with the combination of multi-modal features at different levels of medical images and medical texts, and the interaction between the question and the visual information extracted from the text semantics is often overlooked.

We propose a novel bi-branched model based on parallel network and image retrieval for medical visual question answering (BPI-MVQA). The main contributions of this work can be summarized as follows:

We propose a bi-branched neural network model that can be used in different classification methods for different types of training data for VQA-Med. The first branch uses a model similar to a transformer [13] to extract image features in parallel for classification. The second branch uses the method which retrieves the similarity of images and outputs the labels of similar images as similar text descriptions. Our model achieves state-of-the-art results on three datasets, which proves that our model is effective for VQA-Med.

We propose a novel method that uses the pre-trained VGG16 network [14], which removes the full connection layer to output image features, and then select the answer labels of similar images by calculating the cosine similarity of the feature matrices of the two images. This method significantly improves the accuracy of part of the data on the test set.

We propose the ResNet152 [15, 16] and Gate Recurrent Unit(GRU) [17] parallel structure to extract both full-scale image features and local features. Its purpose is to preserve the spatial feature information of images in different dimensions. Then, the original three-channel images are processed into single-channel grayscale images and input into the stacked GRU network to retain the sequence feature information of the images. Finally, the features extracted from each layer of ResNet152 and the output of the features from the GRU network are concatenated as complete features of the images.

We apply the transformer structure model as the main part of the multi-classification model. In the NLP task of biomedicine, Biobert [18] is much better than Bidirectional Encoder Representations from Transformers(Bert) [19] in many biomedical text mining tasks and is more suitable for biomedical data training because it utilizes the biomedical corpus on PubMed to understand complex biomedical literature. Unlike the traditional Bert model input, we take the concatenated image features and question features as the input of the transformer and make use of their multiple characteristics in the transformer. The multi-head self-attention mechanism fuses the input features, and then the model outputs the answers.

### Related work

The development of VQA-Med is a very interesting challenge, and many new solutions have emerged to handle VQA tasks. Some methods are also applicable to the VQA-Med field. A classical convolution neural network (CNN) pre-trained on ImageNet is usually selected as the image feature extractor, and a recurrent neural network (RNN) or a model of transformer structure is usually selected as the feature extractor. Peng et al. [20] proposed a deep network model based on ResNet152 and long short-term memory (LSTM) that uses the multi-modal factorized bilinear pooling model (MFB) [21] with a ‘co-attention’ mechanism to fuse features. This end-to-end deep learning network can realize learning on images and questions at the same time, and it won first place in the VQA-Med task of the ImageCLEF2018 competition. Zhou et al. [22] put forward a model based on Inception-Resnet-v2 [23] and BiLSTM [24], which won second place in the competition. Yang et al. [25] put forward a model combined with a stacked attention network (SAN) [26] capable of obtaining the local attention information of the image area through multiple iterations, which won third place in the competition. The following year, Zhejiang University’s team [27] proposed a novel model capable of extracting image features from the middle layer of VGG16 and extracting question features using Bert, which won first place in the ImageCLEF2019 VQA-Med task. Kornuta et al. [28] proposed a modular pipeline architecture that utilized transfer learning and multi-task learning. Liao et al. [29] used a knowledge inference methodology called Skeleton-based Sentence Mapping (SSM) and won first place in the ImageCLEF2021 VQA-Med task. Al-sadi et al. [30] used a effective data augmentation technique and won second place in the ImageCLEF2021 VQA-Med task. Zhang at al. [31] proposed a novel conditional reasoning framework for Med-VQA, aiming to automatically learn effective reasoning skills for various Med-VQA tasks. Gong et al. designed a hierarchical feature extraction structure to capture multi-scale features of medical images and won first place in the ImageCLEF2021 VQA-Med task. Xiao et al. [32] fused the semantic features and image features by Multi-modal Factorized High-order (MFH) Pooling and won second place in the ImageCLEF2021 VQA-Med task. Gupta et al. [33] proposed a hierarchical deep multi-modal network that analyzes and classifies end-user questions and then incorporates a query-specific approach for answer prediction. Do et al. [34] present a new multiple meta-model quantifying method that effectively learns meta-annotation and leverages meaningful features to the VQA-Med task. Lin at al. [35] gave a detailed description of the current situation of Medical Visual Question Answering.

**Fig. 1:**
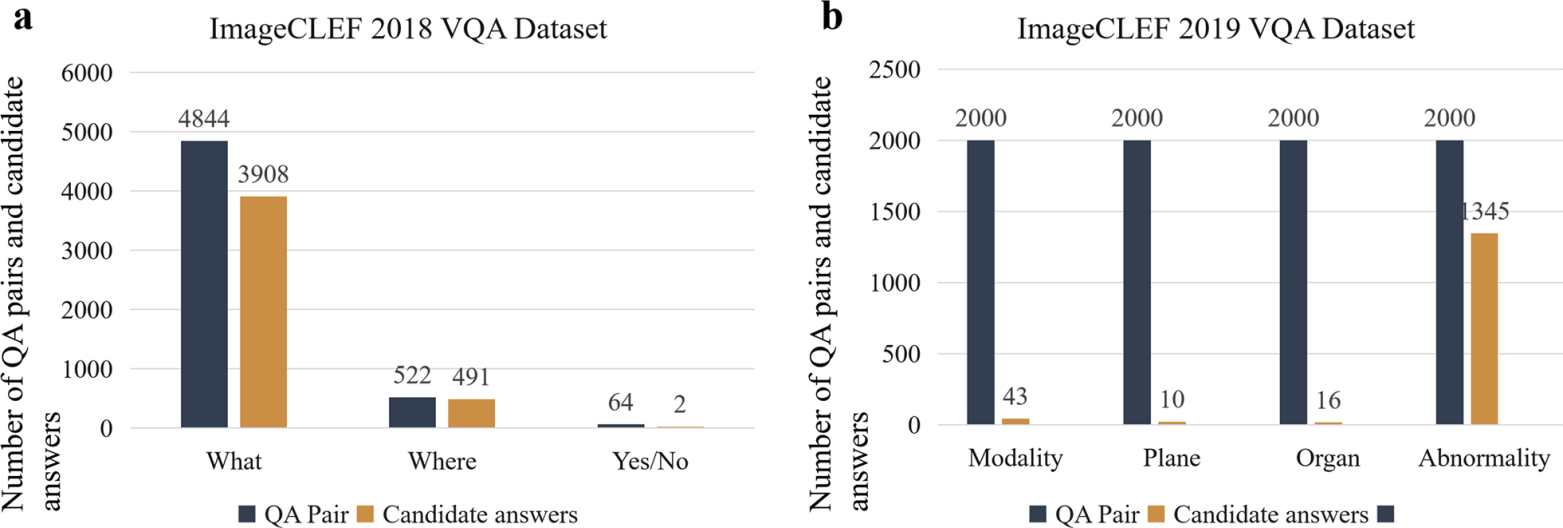
Number of candidate answers on **a** ImageCLEF2018 VQA-Med and **b** ImageCLEF2019 VQA-Med datasets

There are many VQA datasets widely used in general fields, such as COCO-QA [36], VQA-dataset [6], FM-IQA [37], Visual Genome [38], Visual7W [39], and Clevr [40]. Early VQA datasets mainly asked questions about the location, color, and quantity of images. Later, in addition to the simple attributes in the images, some reasoning problems based on common sense were added. At present, the main VQA-Med datasets are ImageCLEF2018 VQA-Med, ImageCLEF2019 VQA-Med, and VQA-Rad. These three datasets are all radiation datasets, and each dataset is divided into several types. The number of question and answer pairs(QA pairs) and the number of candidate answers(candidate answer means the number of different answers contained in all QA pairs )corresponding to each type questions, are shown in Fig. 1.

In addition, data augmentation plays an important role in small sample training. Regarding data augmentation in the field of VQA, Kushal et al. [41] used LSTM to generate a new question sequence corresponding to the original image, and some people translate the questions into other languages and then back into English, these are methods for question data expansion, for image data augmentation, image flipping, rotation at a certain angle, translation, random clipping are frequently used in image processing. Data augmentation [42] can be used to expand a dataset to prevent model overfitting.

## Methods

### Overview of BPI-MVQA

**Fig. 2:**
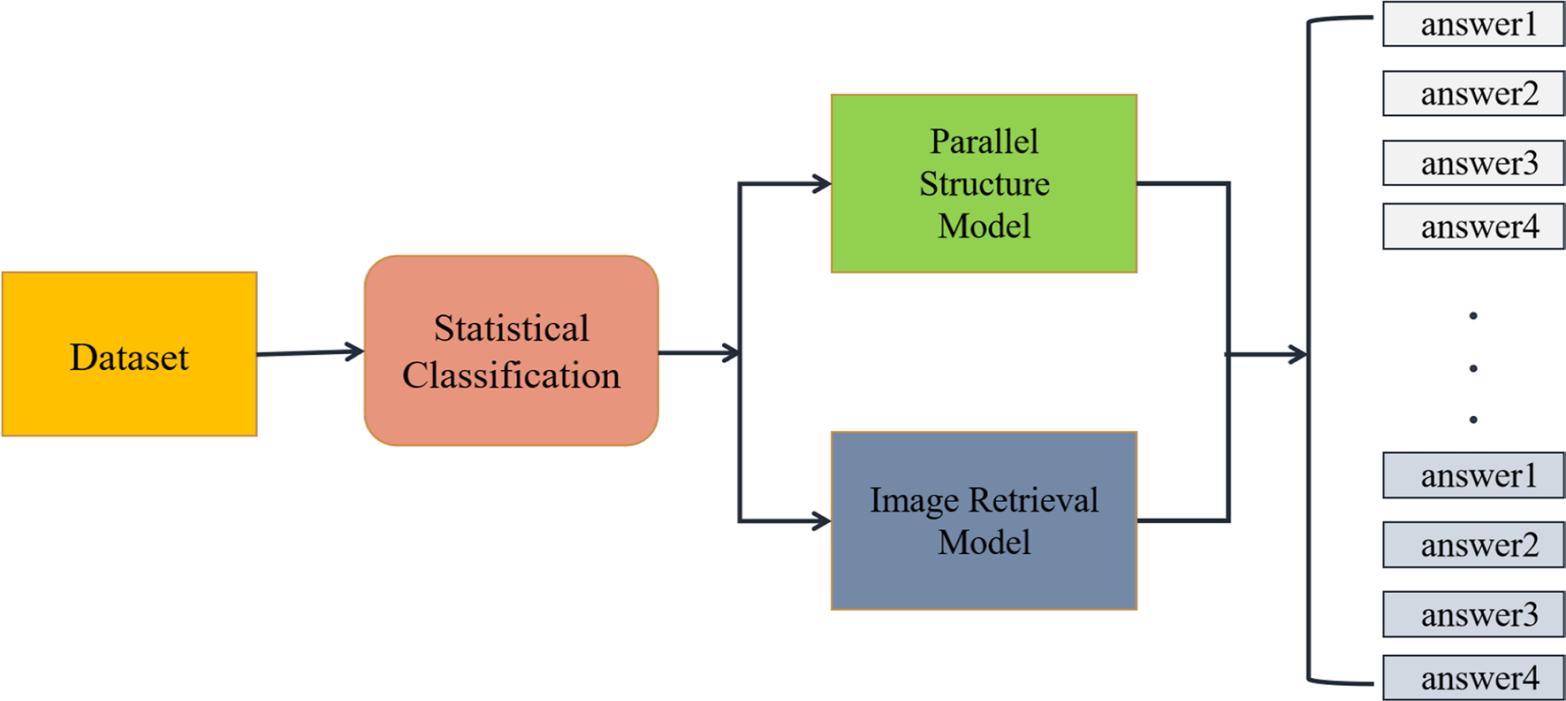
Brief representation of the proposed BPI-MVQA

BPI-MVQA is composed of two branches. We count the number of candidate answers for each type of training set data as the first step. According to Fig. 1, if the current type of data has few candidate answers and is easy to classify, it will be transferred to the first branch(parallel structure model). Otherwise, the data will be transferred to the second branch(image retrieval model). The image extractor in the first branch, which has a transformer structure with a parallel structure, will be used for classification. Next, the pre-trained VGG16 network in the second branch will be used to retrieve the similarity of images and output the labels of similar images as similar text descriptions. The whole process is shown in Fig. 2.

### Parallel structure model

**Fig. 3:**
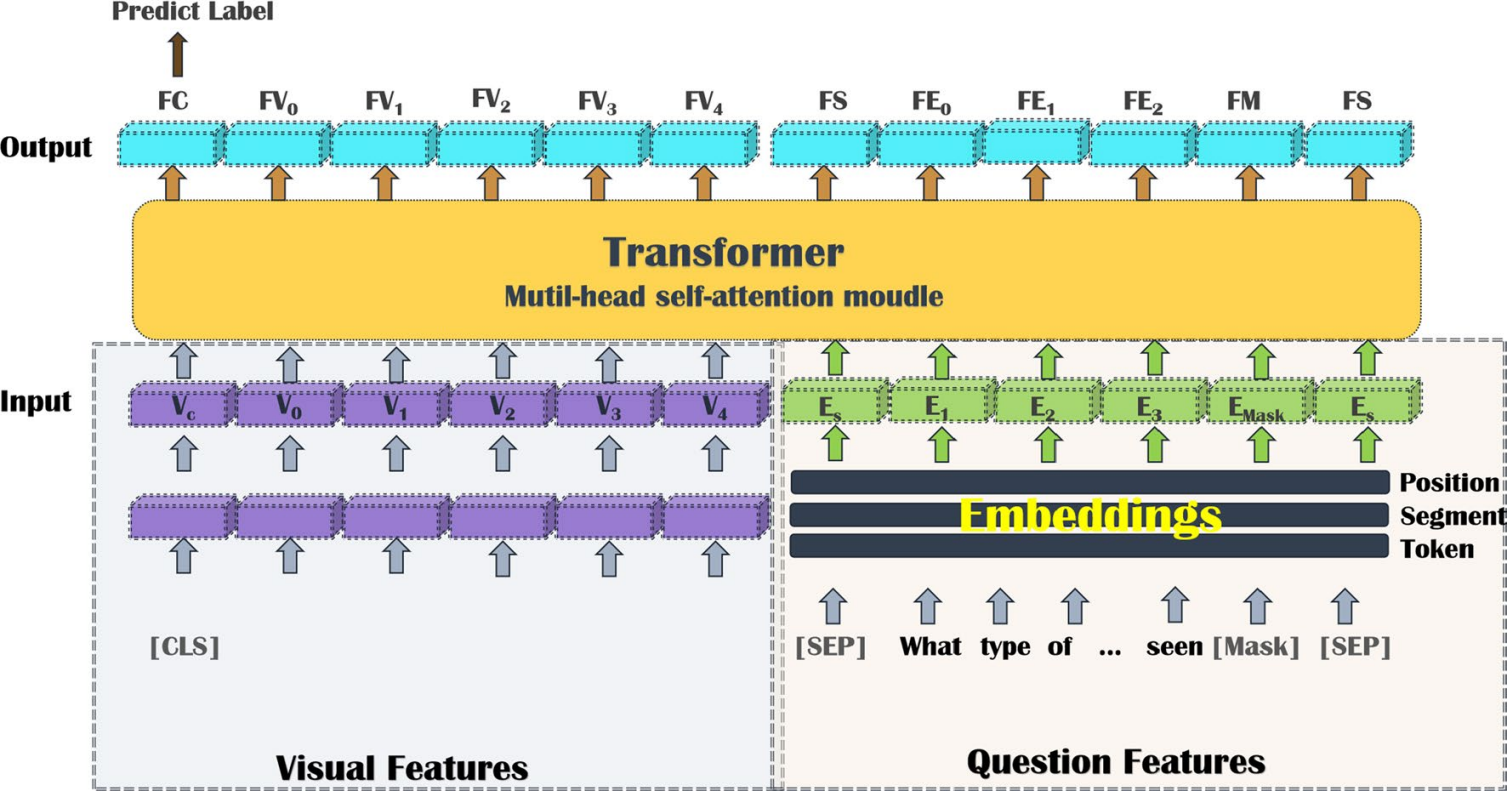
Overall structure of the parallel structure model

In the first branch of BPI-MVQA, we choose the transformer structure as the main framework of our parallel structure model. Different from the VilBert [43] and LXMERT [44] models, which input the questions and images into two independent transformers to process the features of the two parts separately, our model takes the features of the two parts as the input of the single transformer. The idea of the parallel network structure is embodied in the image feature extraction. As shown in the visual features part of Fig. 3, the feature blocks $V_{i}$ are realized by the parallel network structure composed of ResNet152 and GRU. As shown in the question features part of Fig. 3, the feature blocks $E_{i}$ are embedded by three-layer word embedding based on a biomedical corpus. Subsequently, $V_{i}$ and $E_{i}$ are concatenated into a complete visual feature and fused by the multi-head self-attention module of the transformer framework. In addition, the special symbols [CLS] and [SEP] are used to separate sentences. Figure 3 shows the overall structure of our parallel structure model.

#### Image feature extraction

In this parallel structure model, we adopt a parallel network to extract the image features. Firstly, we use an improved CNN model to extract the spatial features of the medical images. Secondly, we use an RNN model to extract the sequence features of the medical images. The following two sections introduce these parts of the parallel network model.

**Fig. 4:**
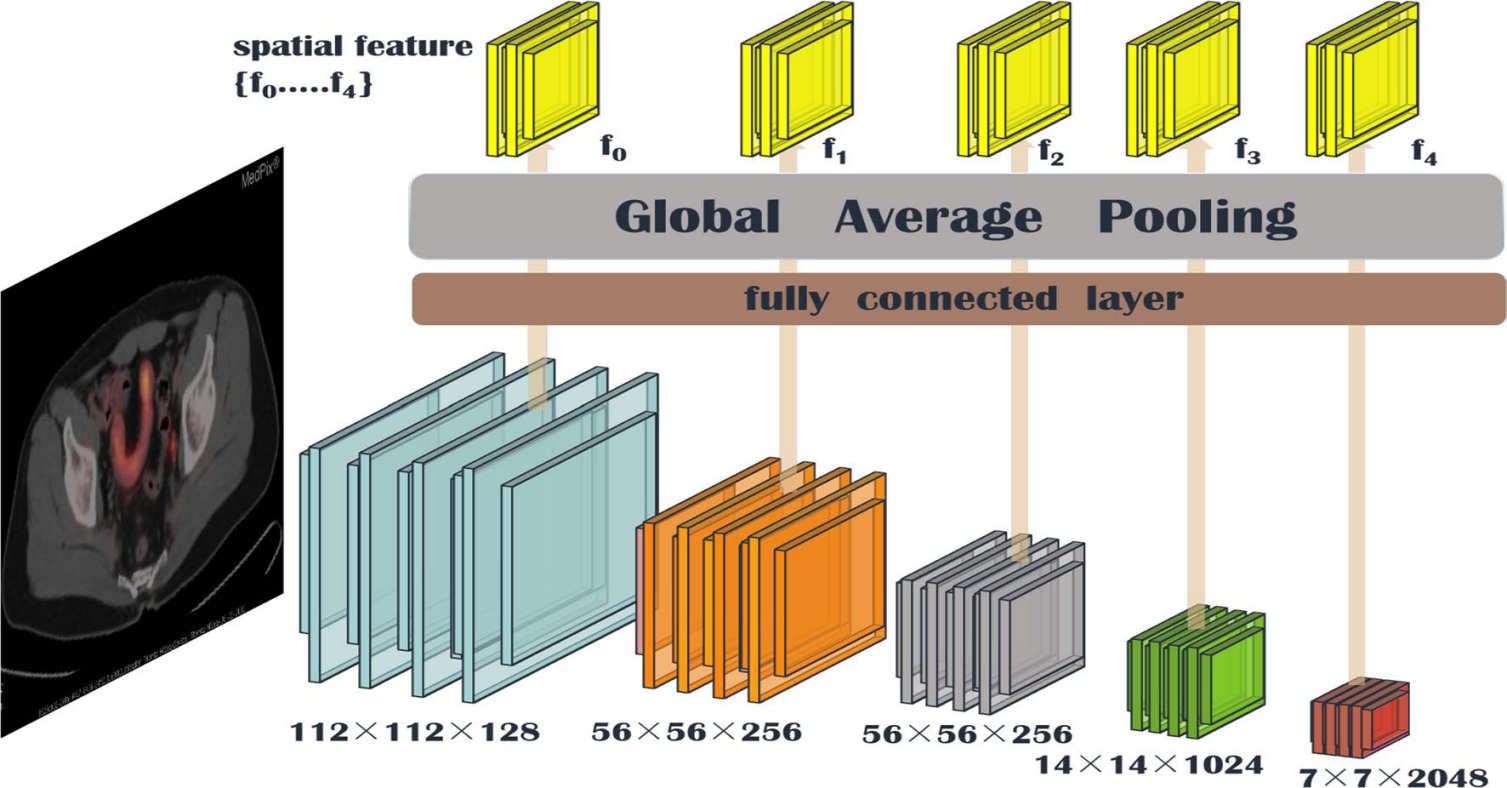
CNN part of the parallel structure model

*CNN part* In the CNN portion of the parallel network model, we use the pre-trained ResNet152 model. We know that the deeper the network is, the more difficult it is to train. There will be problems of gradient disappearance and gradient explosion. Skip connection can be activated from one layer, and then quickly feedback to another layer or even deeper layer, and a residual network can be constructed to train a deeper network with the skip connection. We input the image into ResNet152 after image preprocessing involving processes such as rotation, random resizing, brightness adjustment, and contrast adjustment, and retain the features of images passing through each intermediate layer. Then, the features are passed through the full connection layer, image features are projected into the same dimension space as question features, and the global average pooling (GAP) [45] operation is performed. GAP reduces most of the parameters compared with the fully connected operation while unifying the dimensions to prevent overfitting. The structure of the CNN part of the parallel network model is shown in Fig. 4.

**Fig. 5:**
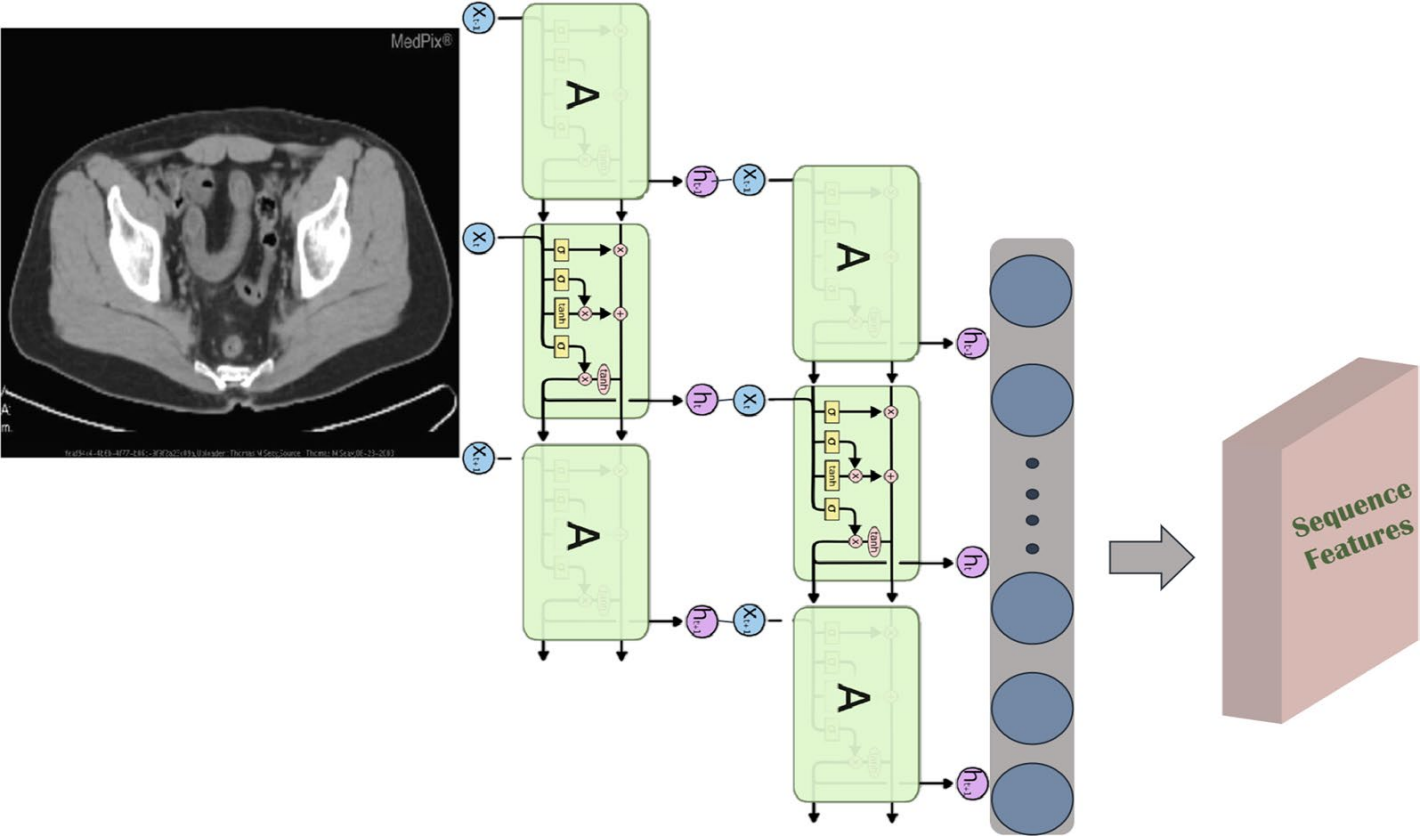
RNN part of the parallel structure model

*RNN part* It has been accepted that images have spatial features. However, if we look at the pixel level, there is also a temporal relationship between each pixel of the image. For example, if we regard the width of an image as the eigenvalue and the height of the image as the time step, we can consider that each row of pixels in each image has a time-dependent relationship. Therefore, when designing the model, we should not only consider the spatial relationship of the image but also the temporal relationship between pixels. We use a two-layer stacked GRU as the RNN module, in which the original three-channel images are processed into single-channel grayscale images and input into the stacked GRU network to retain the sequence feature information of the images. The structure of the RNN part of the parallel network model is shown in Fig. 5.

**Fig. 6:**
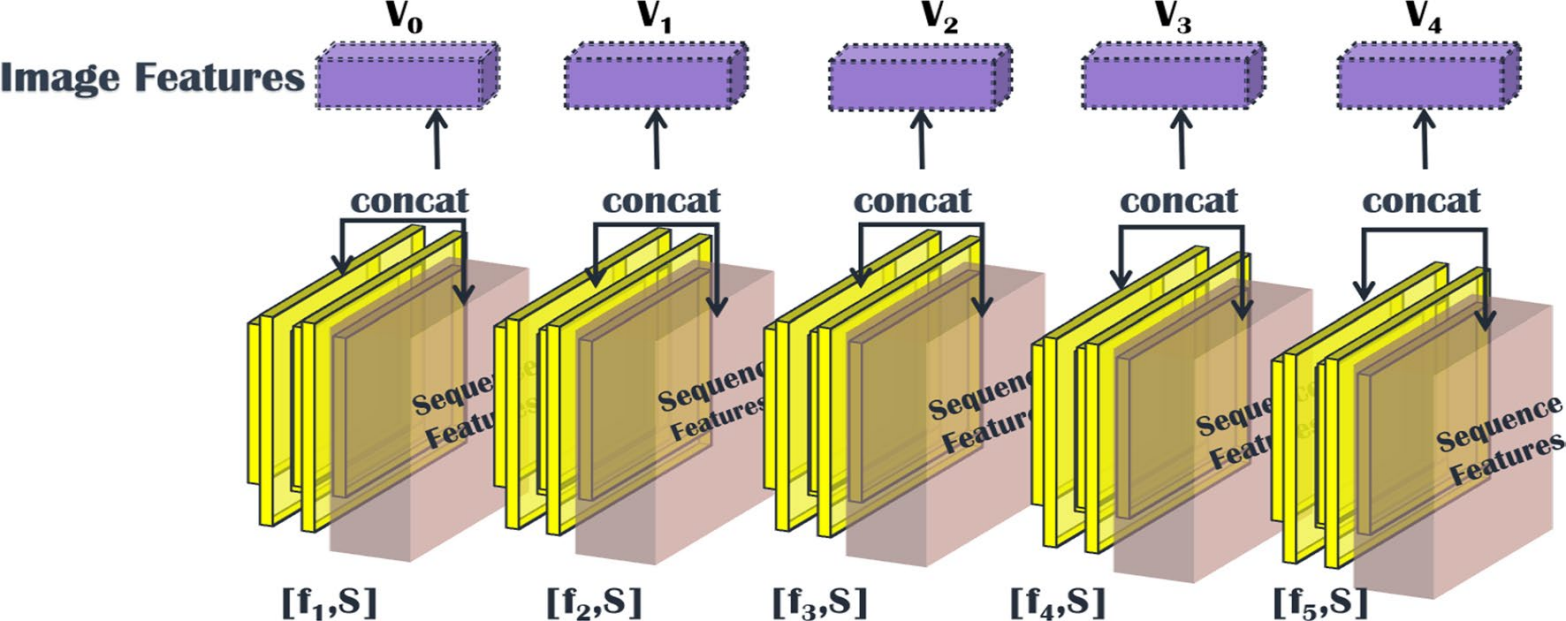
Structure of the image feature fusion module

*Parallel feature fusion* If the image features of different layers are concatenated together and the image sequence information is extracted again through the GRU network, the dimension of the feature matrix will be reduced from a large dimension to a very small dimension, which may result in the loss of many useful image features. Therefore, we combine the image features of different middle layers with the sequence features of the image extracted by GRU to get the feature matrix of the visual portion. The structure of the image feature fusion module is shown in Fig. 6. We take the final output $f_{1}... f_{5}$ as the image features, combine them with the text features *S*, and then input them into the model of transformer structure.

#### Text feature extraction

We convert all questions and answers into lowercase letters to prevent two candidates with the same meaning from being extracted due to different letter cases. Our model adopts three embedding methods in the transformer structure and uses the biomedical corpus based on PubMed. In order to input question features into the transformer structure model, we first use token embedding to transform each word into a fixed dimensional vector. In this process, two special tokens, [CLS] and [SEP], are inserted into the beginning and the end of the input text, respectively, to segment the sentence. We then use segment embedding to assist the transformer in distinguishing the vector representation of two adjacent sentences. Finally, we use position embedding to introduce the coding information of the sequence order with the following formula.

1 $$\begin{aligned} \left\{ \begin{array}{lr} PE_{2i}=\sin (p/10000^{2i/d_{pos}}) &{} \\ PE_{2i+1}=\cos (p/10000^{2i/d_{pos}}) &{}, \end{array} \right. \end{aligned}$$

where *pos* stands for position and *i* stands for dimension. This formula means that for each word vector, the *sin* variable is added at each even position, and the *cos* variable is added at each odd position to fill the whole *PE* matrix. It can be seen from Eq. (1) that each dimension *i* corresponds to a sine or cosine curve of a different period. When *i* = 0, it is a *sin* function with a period of 2$\pi$, and when *i* = 1, it is a *cos* function with a period of 2$\pi$. For two different positions $pos_{1}$ and $pos_{2}$ in one dimension, if they have the same coding value on a certain dimension 2*i*, the difference between the two positions is equal to the period of the curve where the dimension is located, that is, $|pos_{2}-pos_{1}|$ = $T_{2i}$, and for another dimension $2i+1$(2i$\ne$2i+1). Since $T_{2i}$ $\ne$ $T_{2i+1}$, the coded values of $pos_{1}$ and $pos_{2}$ on different dimensions will not be equal. This coding method ensures that different positions will not be coded to exactly the same value in all dimensions.

#### Fusion of question features and image features

**Fig. 7:**
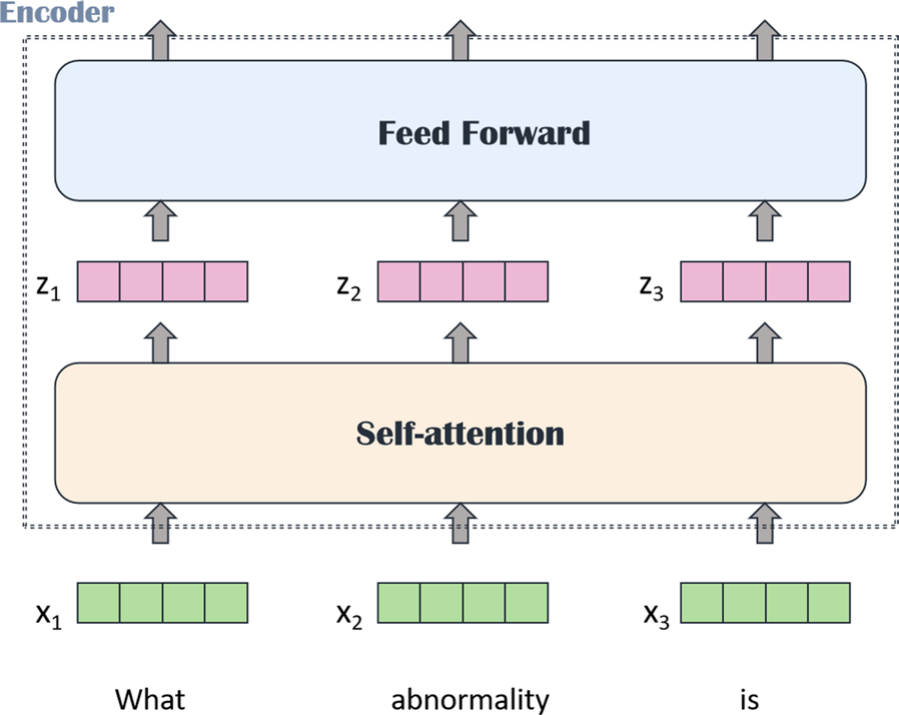
Structure of simple transformer

The transformer is composed of a self-attention module and a feed forward neural network (FFN). It uses the attention mechanism to solve the problem of information loss in the process of sequential computing, as shown in Fig. 7. We embed the above-mentioned image features into the front part of the question features, integrate the two parts of features into a feature matrix, and then input it into the stacked four-layer transformer structure. As a result, the model can learn the dependency between image features and question features, and capture the internal structure of the input feature vector. For example, if the input is a sentence, in order to consider the order of the input feature sequence, we use position embedding to determine the position of each word in the sentence. However, because our medical image lacks the relevant annotation for target detection, we cannot match the local information of the image with the question information in the position. We can only input the overall image features from different layers extracted from the parallel structure model with the word vector into the transformer for attention operation. An advantage of this method, though, is that it pays more attention to the dependence between image and question features than the traditional method of inputting features. We input the joint feature $X_{e}\in R^{n\times d_{model}}$ of the images and the questions into the model. First, a linear transformation is carried out, and then the weight matrices $W_{Q}$, $W_{k}$, and $W_{v}$ are assigned to the corresponding matrices *Q*, *K*, and *V* so as to generate the *Q*, *K*, and *V* matrices. The *Q*, *K*, and *V* matrices of self-attention are $X_{e}W^{Q}$, $X_{e}W^{K}$, and $X_{e}W^{V}$, respectively. Because the weights are different, the final *Q*, *K*, and *V* matrices are different. We get the *Q*, *K*, and *V* matrices using scaled dot-product attention for similarity computation. This *softmax* score determines the possibility of the current word in each word position in each sentence. The following is the formula of the attention mechanism.

2 $$\begin{aligned} Attention(Q,K,V)=softmax\left( \frac{QK^{T}}{\sqrt{d_{k}}}\right) V. \end{aligned}$$

The essence of the multi-head attention mechanism is to independently calculate multiple self-attention mechanisms and then concatenate them, as shown in Eq. 3 and Eq. 4. Equation 5 represents the principle of multi-head self-attention. Each head learns features in different representation spaces. For example, the two heads may notice slightly different emphases, which gives the model more capacity for feature information. We divide the 312-dimensional feature vector into *h* dimensions. It should be noted that *h* must be a factor of 312. Here, we apply *h* = 8 and *h* = 12 to learn the feature differences of 8 and 12 representation spaces, respectively.

3 $$\begin{aligned} head_{i}= & {} Attention(QW^{Q}_{i},KW^{K}_{i},VW^{V}_{i}). \end{aligned}$$

4 $$\begin{aligned} MultiHead(Q,K,V)= & {} Concat(head_{1},...head_{h})W^{o}. \end{aligned}$$

5 $$\begin{aligned} head_{i}= & {} Attention(X_{e}W^{Q}_{i},X_{e}W^{K}_{i},X_{e}W^{V}_{i}). \end{aligned}$$

After the calculation of multi-head self-attention, residual connection and normalization will be carried out, and the result will be sent to the full connection layer for nonlinear transformation to get the final output. The activation function we use is relu. Finally, we perform classification by the location of the output of the special symbol [CLS].

### Image retrieval model

**Fig. 8:**
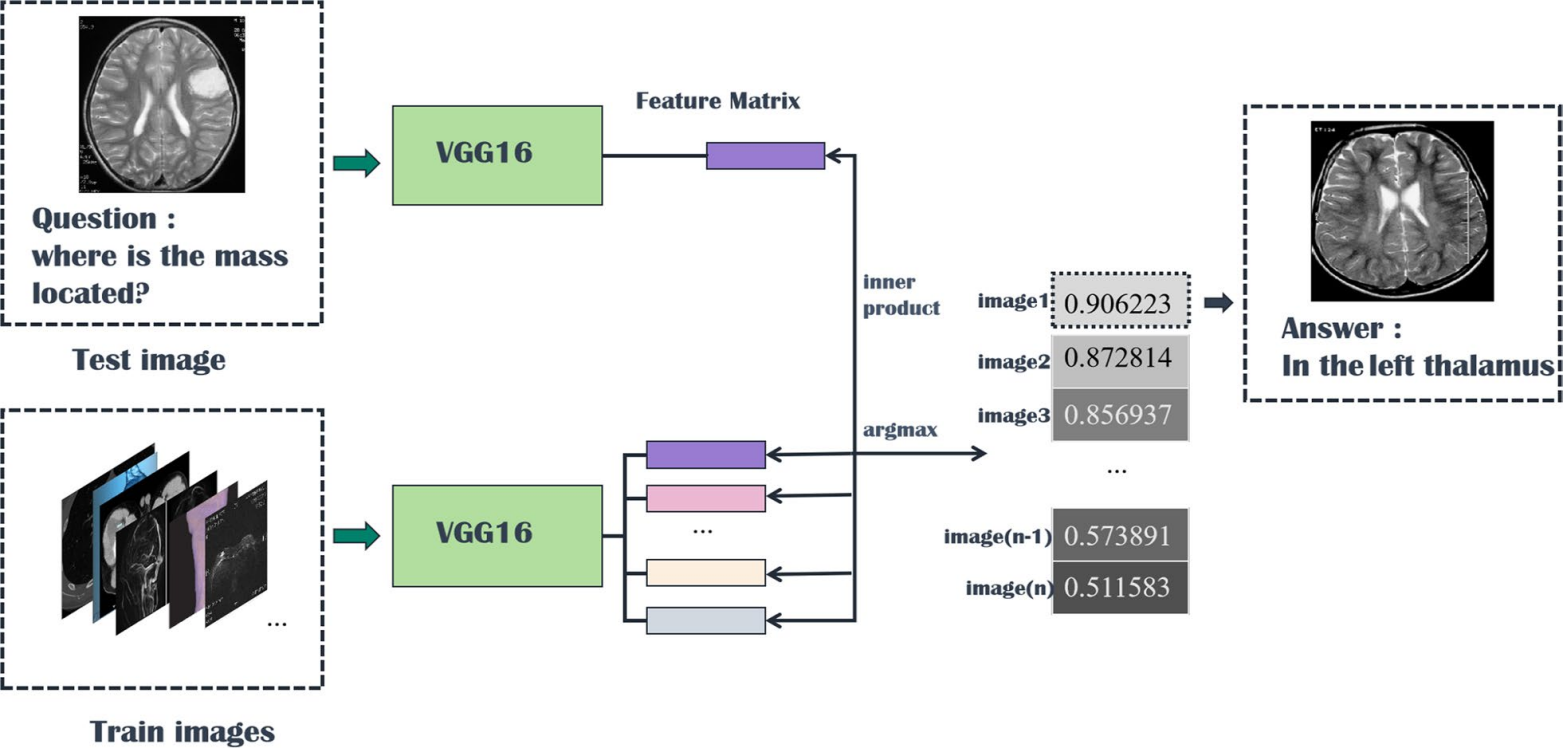
Overall structure of the image retrieval model

**Fig. 9:**
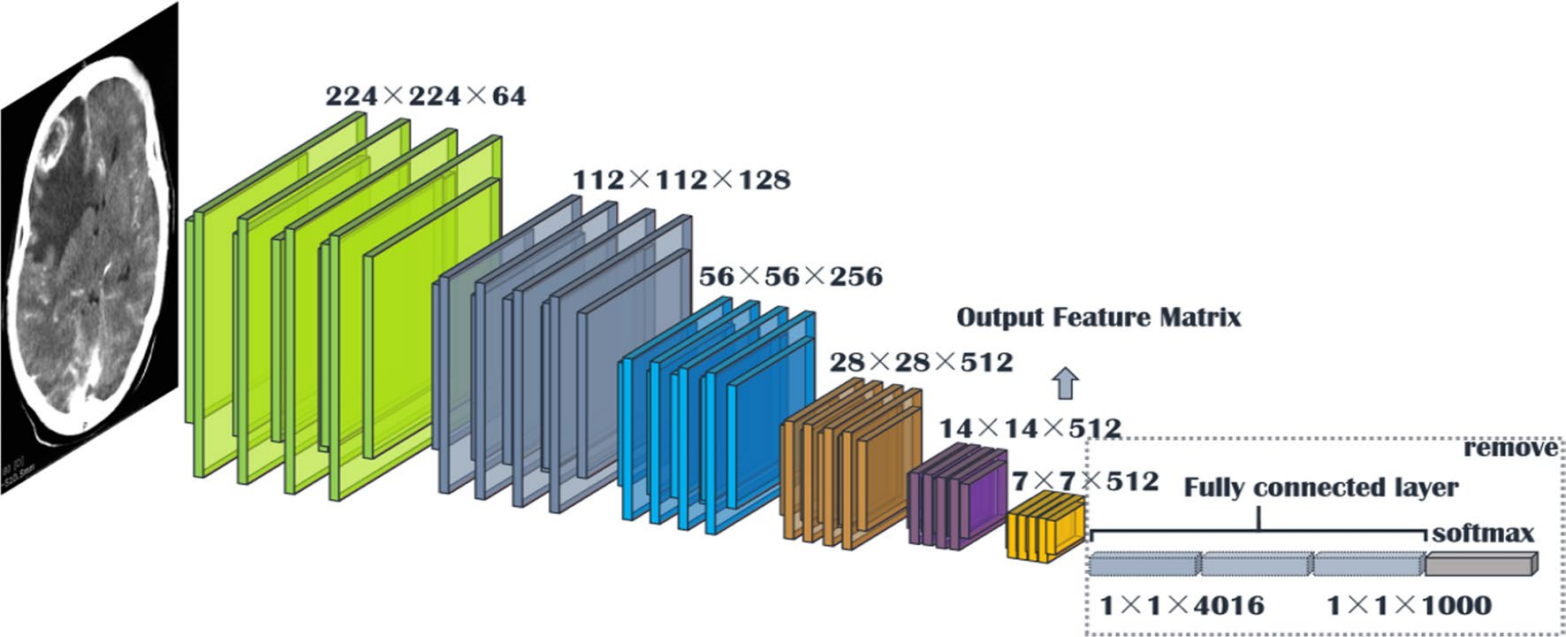
VGG16 structure of the image retrieval model

**Fig. 10:**
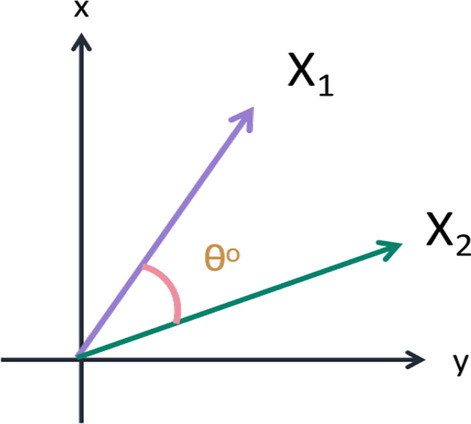
Visual graph and formula of cosine similarity

Inspired by [46], we use the answers of the training set as the labels of the corresponding images, ignoring the influence of question features on the classification results. The main idea is to retrieve the most similar images and labels on the training set with the images to be tested. Figure 8 shows the overall structure of our image retrieval model. VGG16 network is selected to be the feature extractor, because for VGG16, the number of channels in the first layer of the network is 64, and the number of channels in each subsequent layer is doubled. With the increase in the number of channels, more information can be extracted. Next, we remove the fully connected layers and obtain the image feature from the last convolution layer. The structure of the image retrieval model is shown in Fig. 9. We use this method to predict the answers to irregular, open-ended type questions, which have many different answers on the training set of three datasets. Because it is difficult to distinguish effective candidate answers, we start with the analysis of medical image features. First, we input the images of the training set into VGG16, and then we divide the output feature matrix by the matrix’s own normal form as the image features to be compared. The reason for doing this is that the dot product of two matrices is directly proportional to the cosine of the angle between them, so the closer they are in direction, the larger the dot product is, and the higher the similarity between the two images is. The mathematical principle is shown in Fig. 10. As shown in Eq. 6, $X_{1}$ and $X_{2}$ represent the vectors of two features corresponding to Fig. 10, where the $x_{1}$ and $x_{2}$ sets are the elements in the vectors, and $\theta$ is their angle.

6 $$\begin{aligned} \cos (\theta )=\frac{X_{1}X_{2}}{|X_{1}||X_{2}|}=\frac{\sum \limits _{k=1}^{n}x_{1k}x_{2k}}{\sqrt{\sum \limits _{k=1}^{n}x^{2}_{1k}}\sqrt{\sum \limits _{k=1}^{n}x^{2}_{2k}}}. \end{aligned}$$

If the feature matrix of the image to be tested is $A\in R^{n\times n}$, the *a* and *b* sets are the elements in the matrices, and the feature of an image in the training set is $B\in R^{n\times n}$, the inner product of the two matrices, as shown in Eq. 7.

7 $$\begin{aligned} A\bullet B=<A,B>=Tr(A^{T}B)={\sum \limits _{k=1}^{n}\sum \limits _{k=1}^{n}a_{ij}b_{ij}}=(vecA)^{T}vecB. \end{aligned}$$

The similarity of the two matrices is obtained, and then we can output the text description of the image with the highest similarity. Table 1 shows similar images and their corresponding text descriptions. On the left side of the table is the image to be tested, as well as its question and true answer, and on the right side is the image of the training set retrieved with the image retrieval model, as well as its question and answer pair.

**Table 1:** An example of using the image retrieval model

	test image	image retrieved
	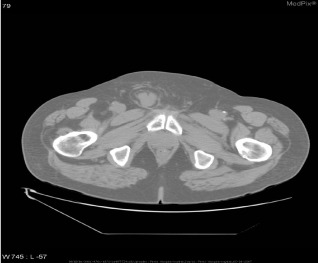	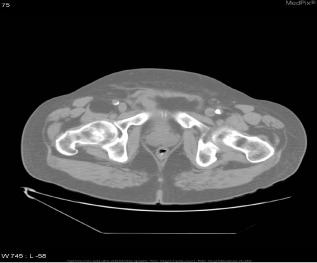
Question	What abnormality is seen in the image?	What is most alarming about this ct scan?
Answer	**Inguinal hernia involving bladder**	**Inguinal hernia involving bladder**

## Evaluation metrics

There are many evaluation methods for VQA-Med tasks. Compared with the long text description image caption task, the answers of VQA-Med are usually short sentences. In principle, the evaluation methods generally used for QA can be used for VQA to compare the similarity between predicted answers and true answers. Based on the datasets we use, in order to make our experimental results referential and meaningful, we follow the ImageCLEF VQA-Med competition and propose three evaluation methods to evaluate the predicted answers. For ImageCLEF2018 VQA-Med, ImageCLEF2019 VQA-Med, and VQA-Rad, the evaluation methods include accuracy, WBSS, and BLEU. The three following sections introduce these evaluation methods.

### Accuracy

This evaluation standard is both the simplest and strictest. The algorithm will only consider an answer to be correct if every word, and the order of every word, is matched between the predicted answer and the true answer. Therefore, the accuracy is either 1 or 0, as is shown in Eq. 8.

8 $$\begin{aligned} Accuracy=\frac{|\{Correctlypredictedanswers\}|}{|\{answers\}|} \end{aligned}$$

### Word-based semantic similarity (WBSS)

WBSS is a method to calculate semantic similarity in the medical field. The ImageCLEF 2018 VQA-Med competition created a word-level semantic similarity measurement method based on Wu-Palmer similarity (WUPS) [47]. There is also a concept called concept node, which is a collection of any word and its own synonyms, as shown in Eq. 9.

9 $$\begin{aligned} Sim(x_{1},x_{2})_{Wu \& p}=\frac{2\times Depth(LCS(x_{1},x_{2}))}{Distance(x_{1},x_{2})+2\times Depth(LCS(x_{1},x_{2}))}, \end{aligned}$$

where $Distance(x_{1},x_{2})$ represents the distance between concept nodes $x_{1}$ and $x_{2}$, and $LCS(x_{1},x_{2})$ represents the minimum public inclusion of concept nodes $x_{1}$ and $x_{2}$. The minimum public inclusion refers to the smallest common parent node of two groups of synonyms; that is, the common synonym finally found by cascading their respective synonyms. $Depth (LCS(x_{1},x_{2}))$ represents the depth of the smallest common inclusion of $x_{1}$ and $x_{2}$; that is, the depth of synonym concatenation. There is also a method similar to WBSS called concept-based semantic similarity (CBSS). As the name implies, CBSS is also a method to calculate semantic similarity. The difference between CBSS and WBSS is that CBSS uses MetaMap [48] to extract biomedical concepts from answers through the pymetamap wrapper [7], the dictionaries of the concepts and predicted answers are established, and the semantic similarity between them is calculated by cosine similarity, which is similar to the principle of similarity calculation mentioned in Eq. 6. This is also the evaluation method commonly used by VQA.

### BLEU

The full name of BLEU is ‘bilingual evaluation underground’, and it is a tool used to evaluate the quality of machine language translation. BLEU uses the snowball stemmer and ‘English’ stop word list of NLTK to delete stop words and calculate the BLEU score. Additionally, it uses the matching rule of n-gram to compare the similarity of N groups of words between the predicted answer and the true answer. Generally speaking, 1-gram represents how many words are correctly predicted, whereas n-gram focuses more on the readability and fluency of predicted answers. The BLEU score is actually an ‘improved version of n-gram’ plus a ‘penalty factor of too short’. The calculation formula of *BP* is expressed as follows:

10 $$\begin{aligned} BP=\left\{ \begin{array}{lr} 1 &{}if\quad l_{c}>l_{s} \\ e^{1-\frac{l_{s}}{l_{c}}} &{}if\quad l_{c}\le l_{s} \end{array}, \right. \end{aligned}$$

where $l_{c}$ represents the length of the predicted answer and $l_{s}$ represents the effective length of the true answer. When the length of the predicted answer is larger than the length of the true answer, the penalty coefficient is 1, which means no punishment. Only when the length of the predicted answer is less than the real answer will the penalty factor be calculated.

## Analysis of datasets

### ImageCLEF2018 VQA-Med

We retrieve the ImageCLEF2018 VQA-Med dataset from the official website. The dataset is divided into a training set, validation set, and test set. We classify this dataset into three types of questions: ‘what’, ‘where’, and ‘yes/no’. The ‘what’ and ‘where’ questions are irregular, open-ended questions, whereas the ’yes/no’ questions are close-ended questions. The numbers of question-answer pairs and corresponding images are listed below.

- The training set consists of 5413 question-answer pairs with about 2278 images.
- The validation set consists of 500 question-answer pairs with about 324 images.
- The test set consists of 500 question-answer pairs with about 264 images.

**Fig. 11:**
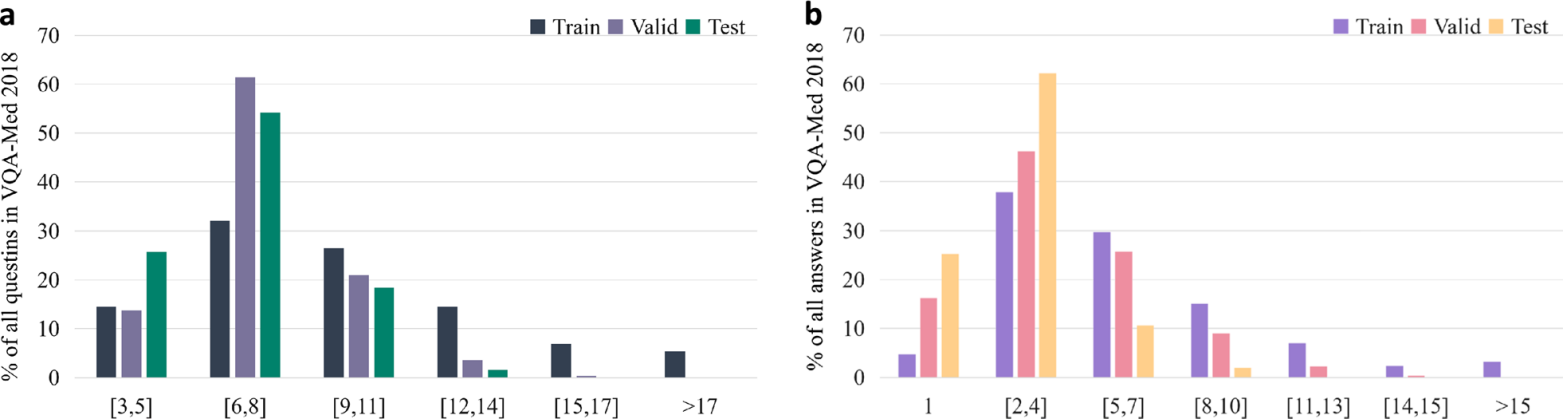
**a** Question word-frequency distribution. **b** Answer word-frequency distribution in the ImageCLEF2018 VQA-Med dataset

The word frequency in the sequences of question and answer pairs in the ImageCLEF2018 VQA-Med dataset is shown in Fig. 11, and the x-axis represents the interval of word frequency.

The word frequency of the questions is concentrated in the interval [3, 12], and the word frequency of the answers is concentrated in the interval [1, 13]. We set the maximum sentence length to 15. Since this dataset is automatically captured from PubMed center papers, one problem is that in addition to radiation images, there are also some 3D reconstruction composite images in the dataset, as shown in Table 2.

**Table 2:** Three examples of the Q&A pair of 3D reconstruction composite images

	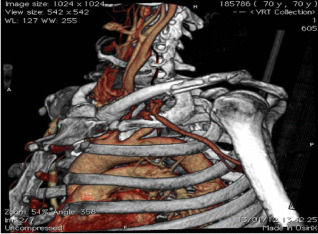	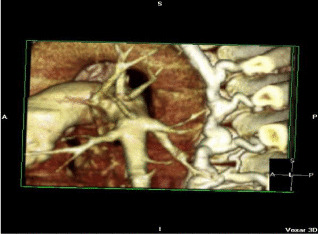	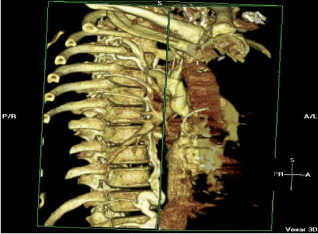
Question	What does ct 3d reconstruction show?	What shows a wispy ill defined embolus in the upper lobar artery?	What shows extensive backfilling of azygous system from superior vena cava?
Answer	Active left subclavian arterial bleeding and the left midshaft clavicular fracture	contrast ct angiogram of left pulmonary artery	contrast ct angiogram

As a result, some meaningless images are mixed into the plane medical radiation image dataset. Another problem is that some part of the dataset is automatically generated from the title of the paper that includes the images, which leads to some questions being inconsistent with the images. These two problems are some of the reasons why almost all experiments based on the ImageCLEF2018 VQA-Med dataset are unsatisfactory. For some reconstructed 3D images, we still process them into a gray-scale image input model in a conventional way, but this will lose the important features of this image and lead to prediction errors.

### ImageCLEF2019 VQA-Med

The ImageCLEF2019 VQA-Med dataset was officially released in 2019, and we registered on the official website to retrieve it. The dataset is divided into a training set, validation set, and test set. The numbers of question-answer pairs and corresponding images are listed below:

- The training set consists of 12792 question-answer pairs with about 3200 images.
- The validation set consists of 2000 question-answer pairs with about 500 images.
- The test set consists of 500 question-answer pairs with about 500 images.

**Fig. 12:**
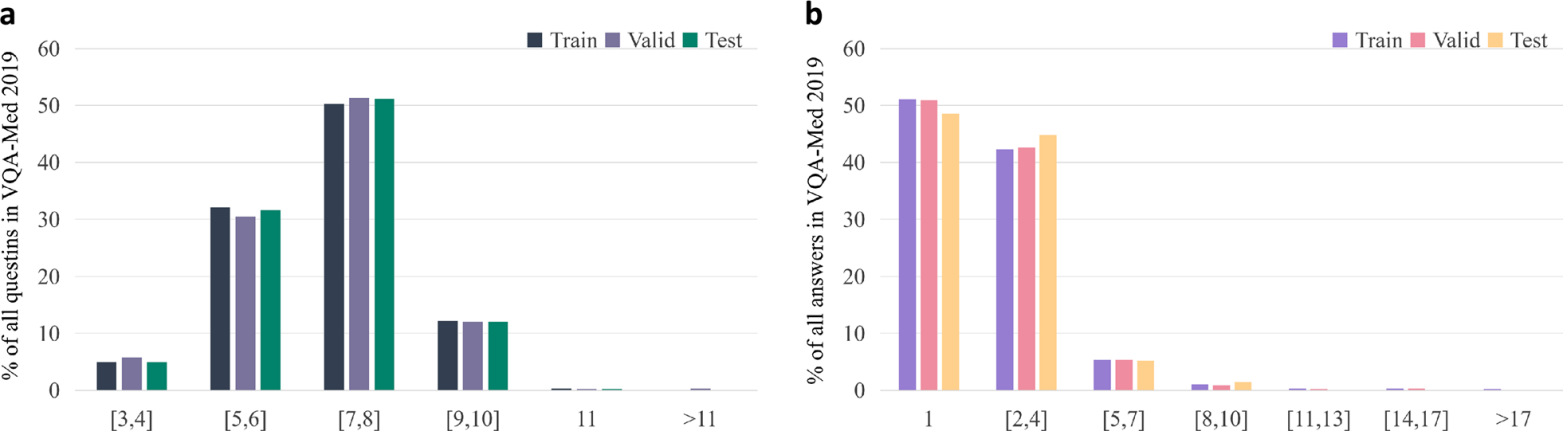
**a** Question word-frequency distribution and **b** answer word-frequency distribution in the ImageCLEF2019 VQA-Med dataset

Medical images in the dataset are balanced samples from Medpix[52], which is an open access case report and teaching case radiology archive. Compared with the ImageCLEF2018 VQA-Med dataset, the ImageCLEF2019 VQA-Med dataset is more standardized and has greater learning value. The official website classifies questions in the ImageCLEF2019 VQA-Med dataset into four types: ‘modality’, ‘plane’, ‘organ’, and ‘abnormality’. Each type of question contains open-ended and close-ended questions, except for the ‘abnormality’ type questions, which only contain irregular, open-ended questions. The word frequency in the sequences of question and answer pairs in the ImageCLEF2019 VQA-Med dataset is shown in Fig. 12, and the x-axis represents the interval of word frequency. The word frequency of the questions is concentrated in the interval [5, 8], and the word frequency of the answers is concentrated in the interval [1, 4]. We set the maximum sentence length to 11.

### VQA-RAD

VQA-RAD is a public medical dataset, which has the same sample source as the ImageCLEF2019 VQA-Med dataset. It only contains a training set and a test set. The numbers of question-answer pairs and corresponding images are listed below:

- The training set consists of 3064 question-answer pairs with about 315 images.
- The test set consists of 451 question-answer pairs with about 315 images.

**Fig. 13:**
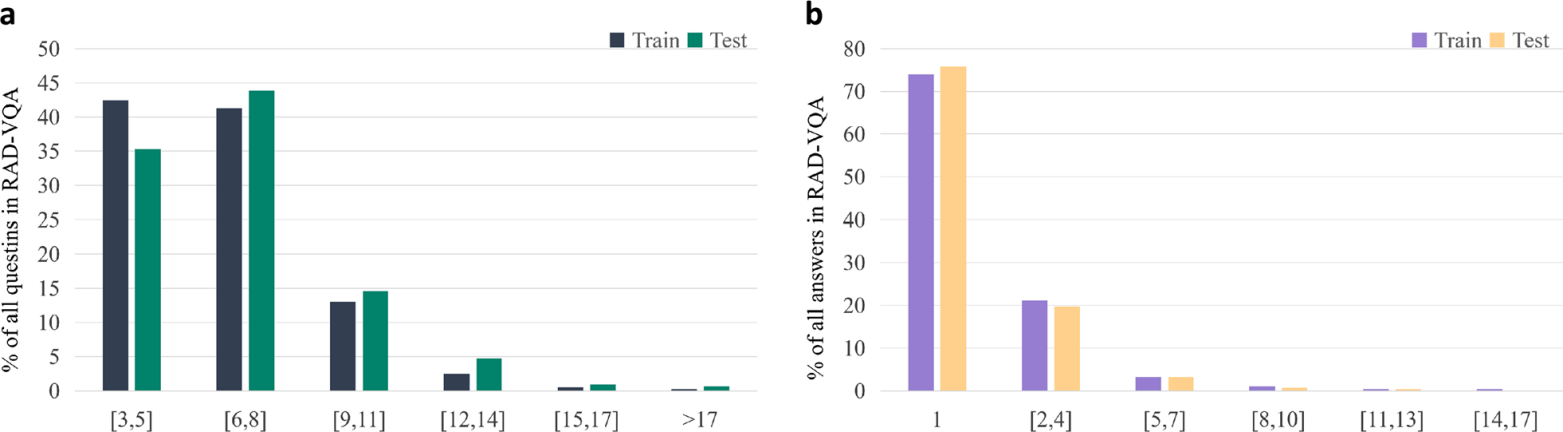
**a** Question word-frequency distribution and **b** answer word-frequency distribution in the VQA-RAD dataset

The VQA-RAD dataset is officially divided into 11 types of questions: ‘modality’, ‘plane’, ‘organ’, ‘abnormality’, ‘pos’, ‘color’, ‘size’, ‘count’, ‘attribute’, ‘other’, and ‘pres’. Each type contains open-ended and close-ended type questions, except for the ‘other’ type questions, which only contain irregular, open-ended questions. The word frequency in the sequences of question and answer pairs in the VQA-Rad dataset is presented in Fig. 13, and the x-axis represents the interval of word frequency.

Because there are many types of data in this dataset, we examine the predicted answers and true answers for each type of question. We find that if the same candidate answers correspond to multiple questions in the training set, and the test images are similar to the images in the training set, the model can probably accurately predict this sample. A further point is that there are many labels that have the same meaning, but they are written differently. For example, there are several ways to write ‘x-ray’, such as ‘Plain film x-ray’ and ‘Xray’, and there are several ways to write ‘T2-MRI’, such as ‘T2 weighted MRI’, ‘MRI - T2 weighted’, and ‘T2’. However, for the sake of rigorous prediction, we have not unified these answers.

## Analysis of experimental results

The training and experiments of the model are based on the machine learning platform of Pytorch and the NVIDIA GeForce RTX 2080Ti graphics card with 11G memory. The model hyper-parameters are shown in Table 3.

**Table 3:** The model hyper-parameters settings

Hyper-parameters	batch-size	iteration	Epoch	Learning rate	Dictionary size
ImageCLEF2018 VQA-Med	64	3213	38	0.0001	3000
ImageCLEF2019 VQA-Med	64	2698	54	0.0001	3000
VQA-RAD	128	885	37	0.0001	3000

According to the statistics of the datasets, we set the dictionary size to 3000 and the longest sentence length to 20. In deep neural network training, batch-size represents the number of samples taken in each training, generally set to a power of 2 to facilitate gradients drop calculation. Limited by the number of samples and hardware computing power, 64 is a more appropriate value. In this paper, different epoch values are determined according to the convergence of the loss function trained by different types of data in each dataset. For example, when training the ‘plane’ type question in the ImageCLEF2019 VQA-Med dataset, the epoch is 54, iteration=number of samples$\times$epoch/batch-size=3200$\times$54/64=2700. Because we trained different types of questions separately, the epoch and iteration in the table are taken as average values. We sets the learning rate to 0.0001 and uses Adam optimizer to dynamically adjust it to ensure that the optimal point is not missed.

The experimental results of the proposed BPI-MVQA on the ImageCLEF2018 VQA-Med dataset are shown in Table 4. For the ‘yes/no’ close-ended type questions, the parallel structure model is used for classification, and the image retrieval model is used to provide the best text description answer to the question. For the irregular, open-ended types of this dataset, the image retrieval model is used to provide the best text description answers to the questions. In order to evaluate the results more comprehensively, we include the conventional recall and precision metrics in the table.

**Table 4:** The experimental results of the proposed BPI-MVQA on the ImageCLEF2018 VQA-Med dataset

Type	WBSS	BLEU	Recall	Precision
What	0.111	0.102	0.014	0.014
Where	0.221	0.21	0.01	0.01
Open-ended	0.136	0.093	0.016	0.02
Close-ended	0.601	0.48	0.346	0.390
All	0.188	0.168	0.012	0.014

Since no candidate answers are exactly the same as the test set answers in the dataset, our prediction can generate predicted answers with similar meanings and reference values based on the image features, as shown in Table 5.

**Table 5:** Three examples of using image retrieval on the ImageCLEF2018 VQA-Med dataset

	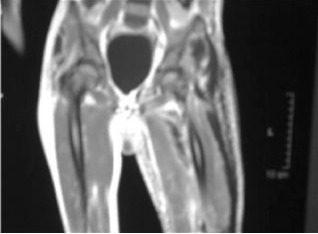	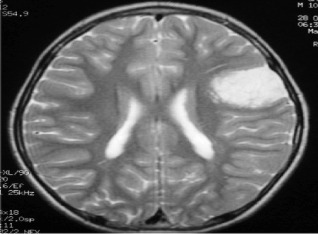	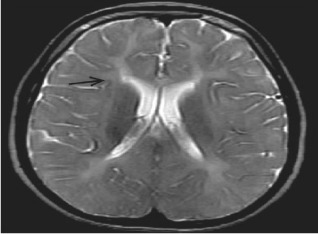
Question	where is the abnormality found?	where is the mass located?	where are the lesions located?
True answer	along the fascia lata	left temporal lobe	in the white matter
Predicted answer	along the synovial lining of the knee joint	in the left thalamus	in the periventricular white matter

The experimental results of BPI-MVQA on the ImageCLEF2019 VQA-Med dataset are shown in Table 6. The parallel structure model is used to predict the answers to the ‘modality’, ‘plane’, and ‘organ’ type questions, and the image retrieval model is used to predict the answers to the irregular, open-ended ‘abnormality’ type questions.

**Table 6:** The experimental results of the proposed BPI-MVQA on the ImageCLEF2019 VQA-Med dataset

Type	Accuracy	BLEU	Recall	Precision
Modality	0.848	0.882	0.645	0.619
Plane	0.848	0.85	0.464	0.407
Organ	0.728	0.759	0.646	0.565
Abnormality	0.192	0.256	0.076	0.079
Open-ended	0.626	0.662	0.187	0.178
Close-ended	0.844	0.851	0.807	0.807
All	0.654	0.687	0.189	0.181

We find that the accuracy of predicted answers to irregular, open-ended ‘abnormality’ type questions has increased significantly. Even if the predicted answer does not match the true answer exactly, our image retrieval method can generate a text description closely related to the true answer, as shown in Table 7. We believe the predicted answer has a certain reference value.

**Table 7:** Three examples of using image retrieval for abnormality type questions in the ImageCLEF2019 VQA-Med dataset

	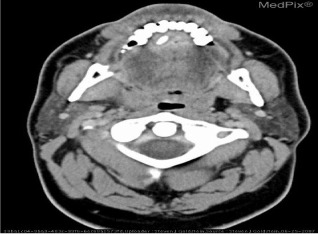	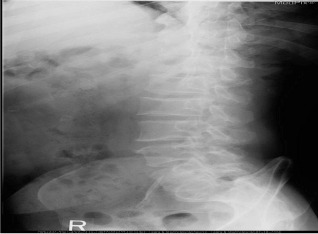	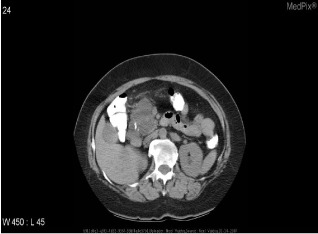
Question	What is abnormal in the ct scan?	What is most alarming about this x-ray?	what abnormality is seen in the image?
True answer	Sialolithiasis, obstructing sialolith	(seronegative) spondylarthropathies	microcystic (serous) adenoma of the pancreas
Predicted answer	Sialolithiasis	spondylarthropathies	hepatic adenoma

In the process of our experiment on VQA-Rad, we encounter the problem of data imbalance. There are only 315 medical images for this dataset, which unsurprisingly leads to a serious underfitting phenomenon-a common problem with small datasets. We perform image augmentation for this dataset, which expands the number of images. This enables us to extract image features from different angles of the same image. Then, we use the image retrieval model to predict the answers to irregular, open-ended ‘other’ type questions. The experimental results show that the predictions of our model on the answers to ‘color’, ‘count’, and ‘attribute’ type questions are completely correct, as shown in Table 8.

**Table 8:** The experimental results of the proposed BPI-MVQA on the VQA-RAD dataset

Type	Accuracy	WBSS	Recall	Precision
Modality	0.714	0.751	0.330	0.295
Plane	0.654	0.696	0.328	0.254
Organ	0.60	0.627	0.467	0.417
Abnormality	0.95	1.0	1.0	1.0
Pos	0.656	0.727	0.481	0.460
Color	1.0	1.0	1.0	1.0
Size	0.804	0.813	0.841	0.800
Count	1.0	1.0	1.0	1.0
Attribute	0.95	1.0	1.0	1.0
Other	0.625	0.684	0.112	0.122
Pres	0.811	0.82	0.538	0.536
Open-ended	0.625	0.624	0.391	0.370
Close-ended	0.841	0.848	0.841	0.840
All	**0.727**	**0.753**	0.397	0.376

**Fig. 14:**
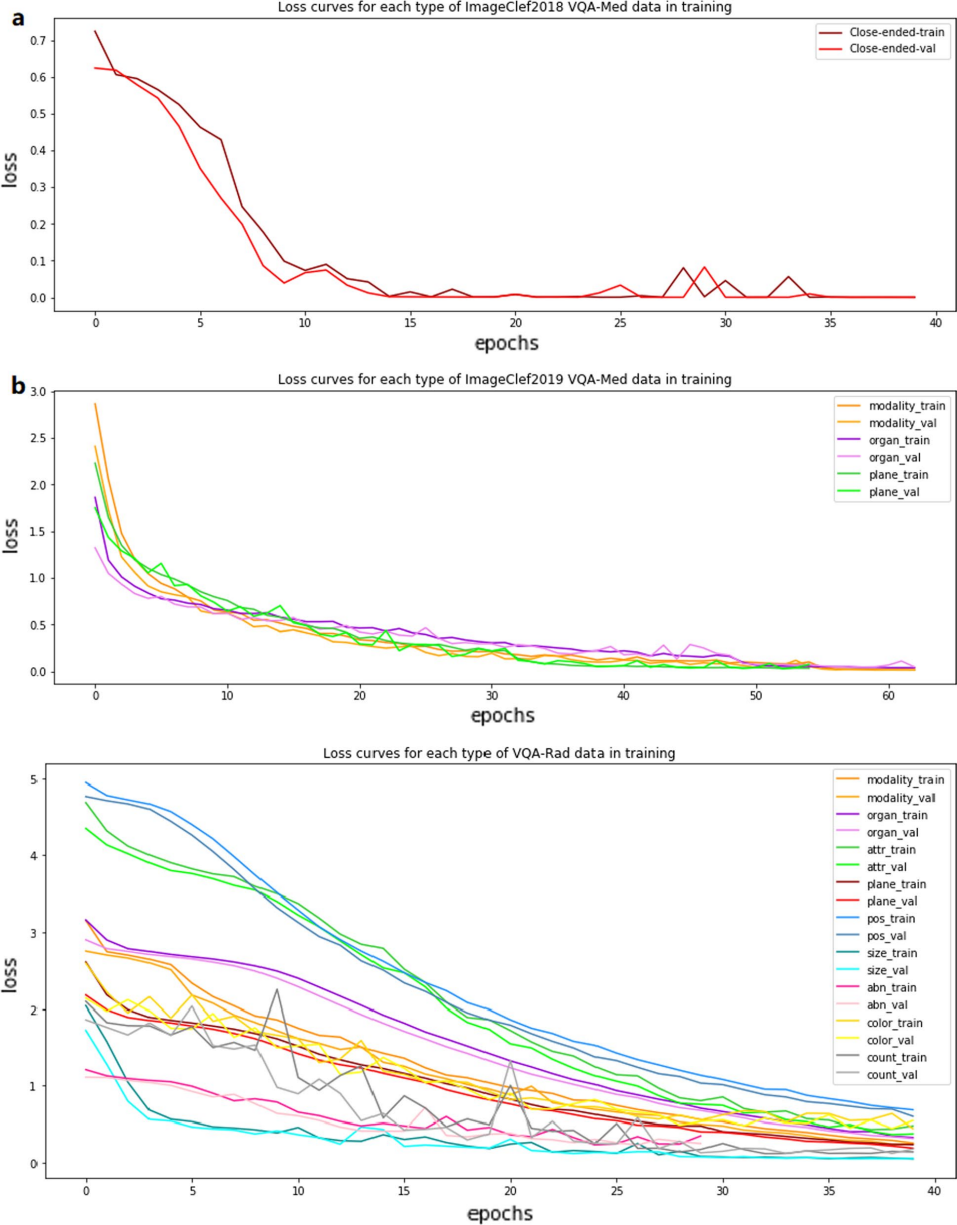
**a** The loss curves of the ImageCLEF2018 VQA-Med dataset. **b** The loss curves of the ImageCLEF2019 VQA-Med dataset. **c** The loss curves of the VQA-Rad dataset

We set different epochs according to different convergence rates, and we use the cross-entropy function as our loss function. Fig. 14 presents the training and validation loss curves of the first branch of BPI-MVQA on the three datasets.

We respectively spend 3 h 10 min, 15 h 25 min, and 6 h 52 min training BPI-MVQA on the ImageCLEF2018 VQA-Med, ImageCLEF2019 VQA-Med, and VQA-RAD datasets. In the real world, the purpose of VQA-Med is to accurately answer clinical problems presented by medical images. We can spend less time on training basic questions, such as questions related to ‘color’ and ‘count’. For example, we only spend 15 minutes on training BPI-MVQA on the type of ‘count’ data, yet it achieves high accuracy. This indicates that we can dedicate more training time to irregular, open-ended type questions. A piece of medical image data contains rich health information about the patient. The VQA-Med system can retrieve this information, which is the key basis for early screening, differential diagnosis, and treatment of various diseases in clinics. The VQA-Med system can also generate health reports to help patients fully understand their condition. In short, VQA-Med has great potential in the medical care industry and service field.

In addition, we conduct ablation experiments to evaluate the effects of innovation points on the performance of BPI-MVQA. Hereafter, W., A., and B. stand for WBSS, accuracy, and BLEU, respectively.

Table 9 shows the effect of using the parallel structure model to extract image features. We compare the effect of including the RNN module (BPI-MVQA) and discluding the RNN module (BPI-MVQA without parallel structure) on the prediction accuracy. The method of combining CNN and RNN structure enriches the information of the feature matrix of each image. In addition, changing the combination of the two parts of features may not only disrupt the regular pattern of spatial features but also fail to reflect the regular pattern of features in sequence. The experimental results show that completely retaining the two parts of feature information is effective. We can see that the efficiency of the model with parallel structure is better than the ordinary model based on ResNet152 and transformer structure.

**Table 9:** Comparison of the model with and without RNN module

model	VQA-Med2018		VQA-Med2019		VQA-RAD	
	W.	B.	A.	B.	A.	W.
BPI-MVQA without RNN module	0.183	0.162	0.626	0.654	0.640	0.673
BPI-MVQA	0.188	0.162	0.654	0.687	0.692	0.753

In the case of irregular, open-ended type questions, the image retrieval model is used to assist model prediction. Table 10 shows the effect of using the bi-branched structure to predict answers. We can see that using this method on a small portion of unbalanced data can improve the accuracy of prediction. We focus this method on the irregular, open-ended type questions of the three datasets, including the ‘abnormality’ type questions of the ImageCLEF2019 VQA-Med dataset and the ‘other’ type questions of the VQA-Rad dataset.

**Table 10:** Comparison of the model with and without image retrieval structure

model	VQA-Med2018		VQA-Med2019		VQA-RAD	
	W.	B.	A.	B.	A.	W.
BPI-MVQA without Image Retrieval	0.153	0.083	0.625	0.660	0.69	0.751
BPI-MVQA	0.188	0.162	0.654	0.687	0.692	0.753

We keep the extraction method of image and question features and the part of image retrieval unchanged, and fuse the two parts of features by traditional point multiplication. The results in Table 11 show that using the transformer to fuse the two parts of features works better.

**Table 11:** Comparison of the fusion of two parts of features with and without transformer structure

model	VQA-Med2018		VQA-Med2019		VQA-RAD	
	W.	B.	A.	B.	A.	W.
BPI-MVQA without transformer fusion	0.183	0.162	0.626	0.654	0.660	0.682
BPI-MVQA	0.188	0.162	0.654	0.687	0.692	0.753

As shown in Table 12, we find the number of heads of the attention mechanism needs to be adjusted in different datasets. When the number of attention mechanism heads is 8, the results of BPI-MVQA on the ImageCLEF2019 VQA-Med dataset are better, but when the number of attention mechanism heads is 12, the results of BPI-MVQA on the VQA-Rad dataset are better. Since the ImageCLEF2018 VQA-Med dataset only uses the transformer structure in the close-ended problem of data, the results do not change much.

**Table 12:** Comparison of 8-head and 12-head attention mechanism

model	VQA-Med2018		VQA-Med2019		VQA-RAD	
	W.	B.	A.	B.	A.	W.
BPI-MVQA with 8-head self-attention	0.184	0.160	0.640	0.644	0.692	0.753
BPI-MVQA with 12-head self-attention	0.188	0.162	0.654	0.687	0.636	0.737

We compare our experimental results with several known models with great experimental results. As can be seen from Tables 13,  14, and 15, our model works best.

**Table 13:** Experimental comparison of BPI-MVQA with other state-of-the-art methods on the ImageCLEF2018 VQA-Med dataset

Model	WBSS	BLEU
ResNet152+LSTM+MFB [20]	0.186	0.158
Inception-Resnet-v2+BiLstm [22]	0.174	0.135
VGG16+SAN(Stacked attention)+LSTM [25]	0.174	0.121
VGG16+BiLSTM+Decision tree classifier [49]	0.053	0.100
BPI-MVQA(Our model)	**0.188**	**0.162**

**Table 14:** Experimental comparison of BPI-MVQA with other state-of-the-art methods on the ImageCLEF2019 VQA-Med dataset

Model	Accuracy	BLEU
VGG16(GAP)+BERT+MFB [27]	0.624	0.644
ResNet152+BERT+Skip-thought vector [50]	0.616	0.634
ResNet152+LSTM+co-attention+MFH [51]	0.566	0.593
SFN [28]	0.558	0.582
GCVMVQA [52]	0.640	0.659
BPI-MVQA(Our model)	**0.654**	**0.687**

**Table 15:** Experimental comparison of BPI-MVQA with other state-of-the-art methods on the VQA-RAD dataset

Model	Accuracy	WBSS
HQS-VQA [33]	0.411	0.437
Inception-Resnet-v2+BiLSTM [22]	0.522	0.532
MEVF+BAN [53]	0.716	-
BPI-MVQA (Our model)	**0.727**	**0.753**

The experimental results show that BPI-MVQA establishes new state-of-the-art results in the three VQA-Med datasets, namely ImageCLEF2018 VQA-Med, ImageCLEF2019 VQA-Med, and VQA-RAD. The main metric scores exceed the best results so far by 0.2%, 1.4%, and 1.1%, respectively. The comparative experiments on three data sets and ablation experiments for each innovation point of the model prove that these ideas proposed in this paper for the VQA-Med task effectively improve the performance. In addition, as shown in Fig. 14, there is no over-fitting or under-fitting in the convergence trend of the loss curve.

## Conclusion

Our life is inseparable from medical treatment, and moving forward the progress of medical care will rely on the accumulation of rich medical cases and doctors’ clinical experience, as well as the assistance of artificial intelligence. VQA-Med is a computer medical assistance system that can help both doctor and patient understand a patient’s current medical status. It can be used to interpret medical images of different organs taken with different imaging modalities and provide information on a variety of diseases. The model we propose herein is a bi-branched structure model. For the regular classification problem, this model uses the features extracted by a hierarchy and the parallel structure of ResNet152 and GRU as image feature extractors, which are combined with three embedding methods and input into the transformer structure with special segmentation symbols. For irregular, open-ended type questions with no effective candidate answers, this model uses image retrieval to give the most similar text description answer to the test image. However, the accuracy of the predicted answers obtained by the image retrieval model is not ideal. Although our model can achieve state-of-the-art performance on three datasets, the results may not be as good on other kinds of datasets. In the first branch of the BPI-MVQA model, image features and text features are simply connected and then input into the transformer structure model, which indicates that we still lack adequate multi-modal feature fusion and need to be improved in the future. So in future work, we may investigate the integration of our proposed model with current innovative and effective methods and models, such as VisualBert [54] and ImageBert [55], which are also transformer structure models that support single-stream input. Furthermore, if we can make a medical image dataset that has the same conditions as the ordinary VQA datasets for target detection, such as the target detection box, our VQA Med system is likely to better align and fuse multi-modal features.

## Data Availability

The ImageCLEF2018 VQA-Med and ImageCLEF2019 VQA-Med datasets are available at https://www.imageclef.org. The VQA-Rad dataset is available at https://www.nature.com/sdata/. Our proposed model is available at https://github.com/liushengyan/BPI-MVQA.

## Abbreviations

VQA-Med: Visual question answering in medical domain
QA: Question and answer
CV: Computer vision
NLP: Natural language processing
CNN: Convolution neural network
RNN: Recurrent neural network
LSTM: Long short-term memory
MFB: Multi-modal factorized bilinear
GRU: Gate recurrent unit
SAN: Stacked attention network
BPI-MVQA: Bi-branch model based on parallel network and image retrieval for medical visual question answering
GAP: Global average pooling
FFN: Feed forword neural network
WBSS: Word-based semantic similarity
BLEU: Bilingual evaluation underground
CBSS: Concept-based semantic similarity
